# Supplementary material for: Patterns of Intron Gain and Loss in Fungi
Source: PLoS Biol. 2004 Nov 30;2(12):e422. doi: 10.1371/journal.pbio.0020422 (PMC532390; doi:10.1371/journal.pbio.0020422)
Supplement: Table S1 — Also available at http://genes.mit.edu/NielsenEtAl/. (4.3 MB ZIP). [file pbio.0020422.st001.zip › NielsenEtAl/html/939.html]

AN4603.1.NCU02296.1.MG10412.1.FG10934.1


```
 CLUSTAL W (1.82) Multiple Sequence Alignments - Introns Inserted


Sequence 1: NCU02296.1	566 aa
Sequence 2: FG10934.1	530 aa
Sequence 3: MG10412.1	550 aa
Sequence 4: AN4603.1	506 aa
Alignment Length: 579 aa
Number Identitical Residues: 251 aa
Alignment Score (without introns) 12639


MG10412.1 	-------MGSQVEPLAVLVSTRAVLTLEDDSLVLSPATITISPNSGKIIAIVPTILPASS
NCU02296.1	MTVLAFLPDAAAEPLVVLVSNRAVLTQPDDSLLLTPATIAISSTTGKIVFVAPTVLSKEA
FG10934.1 	-MASPSTEQYPEGPLSVLVSSRVVVTLPDDSLVTTPASVVVSPVTGKIVSIIPEVLPSSS
AN4603.1  	-------MNGEIPSIAVFTSSRAVIGGR-----LTSATIVVSRTTGKVTAVFDSVIPASD
          	             .: *:.*.*.*:         :.*::.:*  :**:  :   ::. . 

MG10412.1 	FPSSVAYIDQSPKLLLPGLVDAHVHLNEPGRTEWEGFNTGTRAAASGGVTTVIDMPLNAI
NCU02296.1	FPASSTYVDYGNKLILPGLVDAHVHLNEPGRTEWEGFATGTRAAASGGVTTVIDMPLNAI
FG10934.1 	FPAGINYVDHGSKLLIPGLVDAHVHLNEPGRTEWEGFWTGTRAAASGGVTTVVDMPLNAI
AN4603.1  	FPDGTPYTDFSPYVLLPGLVDAHVHLNEPGRTEWEGFYTGTQAAAFGGVTTVIDMPLNAI
          	** .  * * .  :::********************* ***:*** ******:*******

MG10412.1 	PPTTTVANFQEKLKASVGQCWVDVGFYGGVIPGNSQDLLPLVDAGVRGFKGFLIDSG~VD
NCU02296.1	PPTTTLEGFKVKLAAAQGQCWVDVGFYGGVIPGNADQLLPLVEAGVRGFKGFLIESG~VD
FG10934.1 	PPTTTLHGFEEKLRASQGQCWVDVGFYGGVIPGNANELLPLIEAGVRGFKGFLIESG0VD
AN4603.1  	PPTTTVENFKLKLKAAEGKCWVDVGFYGGIIPGNAGELKALVREGVRGFKGFLIDSG0VD
          	*****: .*: ** *: *:**********:****: :* .*:  **********:** **

MG10412.1 	EFPAVSAKDIALAMKALKDAPTTLMFHAEMIPPIADSVGDSVQSSAPPLAPKGALTAYST
NCU02296.1	EFPAVSSSDIALAMTTLKDTPTTLMFHAEMLPPITDSVGDGVTRSEPPLAPKGELTSYST
FG10934.1 	EFPAVSSQDIALAMETLKDSKTTLMFHAEMIPPITQSVGDTVQASEAPLAPSGQLDAYKT
AN4603.1  	EFPAVSSEDVRKAMAELADEPTTLMFHAEMVPPKTP-------SELPEVMPEGAPEAYST
          	******:.*:  **  * *  *********:** :         . . : *.*   :*.*

MG10412.1 	FLESRPPEFETCAVSEILSMTDLAPDLHLHIVHLSATECIPLLREARRRGVNVTAETCFH
NCU02296.1	FLESRPPAFETYAIEEILSMAGLAPQLHLHIVHLSATEAIPILRNARARGVNITAETCFH
FG10934.1 	FLESRPPAFETYAVEEILSQAHIAPQLHLHIVHLSATQCIPLLKAARQSGINITAETCFH
AN4603.1  	FLASRPSEYELCAVEEILSLSHLAPKLPLHIVHLSAMEAIPLLRKARAEGVPITAETCYH
          	** ***. :*  *:.**** : :**.* ******** :.**:*: **  *: :*****:*

MG10412.1 	YLGLASDDIEDGDTRHKCCPPIRSKTNQDGLWSELVRADQHDGVIKTVVSDHSPCTPELK
NCU02296.1	YLGLASDNIEDGDTRHKCCPPIREQSNQDKLWAELTAG--DDSCIRTVVSDHSPCTPELK
FG10934.1 	YLGLTAEEIEKGDTRHKCCPPIREGKNRDGLWEELVA---EDSCIRTVVSDHSPCTPQLK
AN4603.1  	YLSLAAEEIRDGDTRHKCCPPIRSKSNQDALWAELDRH-AEDGVIKTIVSDHSPCTPDLK
          	**.*::::*..************. .*:* ** **     .*. *:*:*********:**

MG10412.1 	LLPNRLAPPP----------ARPPFPHHSDSGIDVSFSP------TEKNKKLEASRRSSG
NCU02296.1	LLPDHLSVQPEFDEQNGALLRRPSLRFT-DSGIDVTLPEDLEEQNTNKTDCCGNHEHAAE
FG10934.1 	LLPQHLETA------------RPDLPHN-DSGIVIPAPE---------------------
AN4603.1  	LLPSHIPGN---------------CSHG-SSKHANTTP----------------------
          	***.::                    .  .*    . .                      

MG10412.1 	SSSSDAPAPVQHTAQGDFFAAWGGISSVGLGLPILHTIATRRARQG------LEAPTIVD
NCU02296.1	LSYVQQDAAAGPQGQGDFFAAWGGISSVGLGLPILHTTATTRG----------APLSIVD
FG10934.1 	----MENPVAEGKGRGDFFAAWGGISSVGLGLPILHSAAKKRADF-------SKTPSITD
AN4603.1  	-----------VVNEGSFLSAWGGISSVGLGLPILWTELSRRKGLTSSPDDTTTKQALQD
          	              .*.*::*************** :  . *    ::...:    :: *

MG10412.1 	MVRLTSQATAKQVGLAHRKGGLKVGMDADVCVFDDAAEWALRSGDMRWKNKCSPWEGHEF
NCU02296.1	VVRLCCQATAKQVGLSHRKGGLKVGMDGDVCVFDDEEEWVLRAGDMKWKNKCSPWEGHQF
FG10934.1 	IVRLCCQATAVQVGLAHRKGAIKVGMDADICVFDDTEEWTFTQGDMRWKNRCSPWEGHEF
AN4603.1  	IVHLCCANTAAQVGLHKSKGDLVPGYDADFCVFDDTAEWEVEPSTMLFRNKCSPYQGRTM
          	:*:* .  ** **** : ** :  * *.*.*****  ** .  . * ::*:***::*: :

MG10412.1 	KGRVLETWVRGHKVFEYGGKNGGFVGGEPVGEAIVEKRTA
NCU02296.1	KGRVRETWLRGTKVYQLGGGNGGFVSGVPVGEAITERRVV
FG10934.1 	TGRVKETWLRGNKVFELGAPNAGFVVSKPVGEPITEKRTV
AN4603.1  	RGMVRETWLRGEKIFNR---DDGFTVKAPSGSLLLEKRV-
          	 * * ***:** *:::    : **.   * *. : *:*.
```
